# Modified string test to improve and confirm by molecular characterization for bacterial identification

**DOI:** 10.1099/acmi.0.000965.v3

**Published:** 2026-06-25

**Authors:** Muhammad Dawood Mian, Saadullah Jan Khan, Rehana Rani, Sara Sadiq, Laila Jafri, Bushra Jamil

**Affiliations:** 1 Department of Life Sciences, Abasyn University Islamabad Campus, Islamabad, Pakistan; 2 BJ Micro Lab, Gulzar Quaid, Rawalpindi, Pakistan; 3 Research Institute for Health Sciences, Chiang Mai University, Chiang Mai 50200, Thailand; 4 Department of Biological Sciences, Faculty of Life Sciences, Health Services Academy, PM Health Complex, Park Road, Chak Shahzad, Pakistan

**Keywords:** bacterial identification, Gram staining, modified string test, potassium hydroxide (KOH), rapid diagnosis

## Abstract

Rapid and reliable identification of bacteria is essential in clinical and environmental microbiology. Gram staining remains a widely used method for preliminary classification; however, it may require additional steps and can be difficult to interpret under certain conditions. To address these limitations, we evaluated a modified potassium hydroxide (KOH) string test incorporating methylene blue to enhance visualization of DNA release and facilitate differentiation between Gram-positive and Gram-negative bacteria.

A total of 185 samples from clinical and environmental sources (including hospitals, soil, salt mines and honeycombs) were analysed. Samples were first assessed using Gram staining and subsequently tested using the modified KOH method. In this procedure, a 3% KOH solution was applied to bacterial colonies, followed by the addition of methylene blue to improve visualization of filament formation.

All Gram-negative isolates demonstrated visible string formation, while Gram-positive isolates showed no string formation, indicating complete concordance with Gram staining results within the tested dataset. Selected isolates were further examined using 16S rRNA gene analysis to support taxonomic identification.

The modified KOH string test provides a rapid, simple and low-cost approach for preliminary bacterial differentiation. While it does not replace conventional or molecular identification methods, it may serve as a useful complementary tool, particularly in resource-limited or high-throughput laboratory settings.

## Data Summary

The authors confirm that all supporting data, code and protocols have been provided within the article or through supplementary data files.

## Introduction

Accurate and timely identification of bacteria is fundamental to microbiology, with important implications for clinical diagnostics, environmental monitoring, public health and agriculture. Rapid and reliable identification is particularly critical in clinical settings, where delays can affect treatment decisions and patient outcomes. Traditional bacterial classification methods rely on morphological and biochemical characteristics, which provide essential baseline information but may be limited in speed, specificity and reliability. These limitations become more pronounced when distinguishing closely related or morphologically similar species, highlighting the need for more efficient and interpretable diagnostic approaches [1,2].

Gram staining, developed by Hans Christian Gram in the late 19th century, remains a fundamental method for differentiating bacteria into Gram-positive and Gram-negative groups based on cell wall structure. While it provides rapid preliminary classification, its interpretation can be influenced by factors such as culture age, staining technique and cell wall integrity. As a result, some bacteria may exhibit ambiguous or Gram-variable staining, complicating classification and requiring additional confirmatory testing [3]. These limitations can delay downstream identification and decision-making, particularly in clinical microbiology, where timely and reliable differentiation is important for guiding appropriate treatment strategies and reducing the risk of inappropriate antimicrobial use [4,5].

To overcome the limitations of conventional methods, molecular diagnostic techniques such as 16S rRNA gene sequencing and MALDI-TOF MS have been widely adopted. These approaches enable precise bacterial identification by analysing genetic or proteomic signatures, with MALDI-TOF MS allowing rapid, high-throughput, species-level differentiation based on protein profiles [6,8]. However, despite their accuracy and efficiency, these methods require specialized equipment, trained personnel and substantial financial investment. As a result, their routine use may be limited in resource-constrained laboratories or for rapid preliminary screening, where simpler and more accessible approaches remain essential [3,9].

While molecular diagnostics such as MALDI-TOF MS provide rapid and precise identification, their high cost and technical requirements limit accessibility in resource-constrained settings. In such contexts, simpler biochemical methods remain important for preliminary bacterial differentiation. The potassium hydroxide (KOH) string test is a widely used biochemical assay that differentiates Gram-negative from Gram-positive bacteria based on cell wall structure. When exposed to 3% KOH, Gram-negative bacteria undergo lysis and release cellular DNA, resulting in the formation of a viscous ‘string’, whereas Gram-positive bacteria do not exhibit this reaction. However, interpretation of the KOH string test can be challenging in some cases due to limited visibility of the filament, leading to potential variability in results. To address this limitation, the present study proposes a modified KOH string test incorporating methylene blue to enhance visualization of DNA release. This modification improves the clarity of filament formation, facilitating easier interpretation of results. As a simple and low-cost enhancement, the method may serve as a practical complementary tool for rapid preliminary differentiation, particularly in settings with limited resources [10,11].

To evaluate the performance and applicability of the modified KOH string test, we analysed 185 samples collected from diverse clinical and environmental sources, including hospitals, soil, salt mines and honeycombs. Each sample was cultured and assessed using both conventional Gram staining and the modified KOH test. The results showed that all isolates classified as Gram-negative by Gram staining exhibited visible string formation in the modified KOH test, whereas Gram-positive isolates showed no string formation, indicating complete concordance between the two methods within the tested dataset. In addition, a subset of isolates was subjected to 16S rRNA gene amplification to support taxonomic identification and provide further confirmation of the observed results [10].

Our findings suggest that the modified KOH string test provides a practical and low-cost approach for preliminary bacterial differentiation, particularly in resource-limited or high-throughput settings. The incorporation of methylene blue improves the visibility of filament formation, facilitating clearer interpretation and reducing ambiguity in test outcomes. Rather than replacing conventional techniques, the modified KOH test is best considered as a complementary tool that can support rapid initial classification prior to further biochemical or molecular analysis. Its simplicity and minimal resource requirements make it applicable across a range of settings, including clinical, environmental and agricultural laboratories. The integration of such modified biochemical approaches with established methods, including molecular techniques such as 16S rRNA gene analysis, may contribute to more efficient and accessible workflows for bacterial identification.

## Methods

### Study design and sample collection

Samples were collected from microbiology laboratories in Rawalpindi and Islamabad, Pakistan, and all laboratory analyses were conducted at BJ Micro Lab (Private) Limited. The study included both clinical and environmental samples to ensure diversity. A total of 152 clinical samples were obtained from multiple hospitals between March 2022 and September 2022. No identifiable patient information was recorded, and all samples were fully anonymized prior to analysis. In addition, 53 environmental samples, including soil, fish and honeycombs, were collected from community sources to provide a broader representation of non-clinical bacterial isolates.

All samples were transported to BJ Micro Lab for processing, purification and identification. Samples were inoculated onto appropriate culture media according to sample type: urine samples on cystine–lactose–electrolyte-deficient agar; pus, nasal and high vaginal swabs on MacConkey and blood agar; sputum samples on blood agar, MacConkey agar, Lowenstein–Jensen medium and chocolate agar; tracheal tube samples on blood and MacConkey agar; and stool samples on deoxycholate citrate agar, *Salmonella*–*Shigella* agar, xylose lysine deoxycholate agar and MacConkey agar. All inoculated plates were incubated aerobically at 37 °C for 24–48 h. Blood culture samples were initially incubated for 24 h in brain heart infusion broth, followed by subculturing onto MacConkey and blood agar. Preliminary identification of bacterial isolates was performed using colony morphology, Gram staining and standard biochemical tests including catalase, oxidase and coagulase tests, along with Analytical Profile Index (API) analysis.

### Gram staining procedure

Gram staining was performed as a reference method for classifying bacterial isolates as Gram-positive or Gram-negative. The standard Gram staining procedure was followed, involving application of crystal violet as the primary stain, iodine as a mordant, alcohol for decolorization and safranin as a counterstain [12]. After staining, Gram-positive bacteria appeared purple, while Gram-negative bacteria appeared pink/red under the microscope.

### Microscopy

Following Gram staining, smears were examined under a light microscope to determine Gram reaction and assess bacterial morphology. Initial observations were made using 10× and 40× objectives to evaluate staining quality, followed by detailed examination under the 100× oil immersion objective. Immersion oil was applied to improve resolution during high-magnification observation.

### Biochemical identification

Biochemical identification of selected Gram-negative bacterial isolates was performed using both conventional biochemical tests and the API 20E system. The API 20E kit comprises a standardized panel of biochemical assays designed for the identification of Enterobacteriaceae and other Gram-negative bacteria. All tests were conducted according to the manufacturer’s instructions. Following incubation, results were interpreted using the API profile system, and bacterial identification was determined by matching the observed biochemical profile with the corresponding database entries [13].

### Modified string test

Following purification on nutrient agar, bacterial colonies were subjected to the modified KOH string test to determine Gram reaction. A small portion of a bacterial colony was transferred using a sterile inoculating loop onto a clean glass slide. One drop of 3% KOH solution was added, and the mixture was gently stirred for ~60 s. After mixing, one drop of 1% methylene blue was added to the preparation to enhance visualization of filament formation. A result was considered positive when a visible viscous filament (‘string’) of ~≥5 mm in length was formed upon lifting the mixture with the inoculating loop. Only distinct filament formation was considered positive, whereas the absence of a visible string was considered negative. Results were interpreted within 1–2 min of KOH application; increased viscosity without continuous filament formation was not considered positive.

## Results

### Growth of culture media

**Fig. 1.**
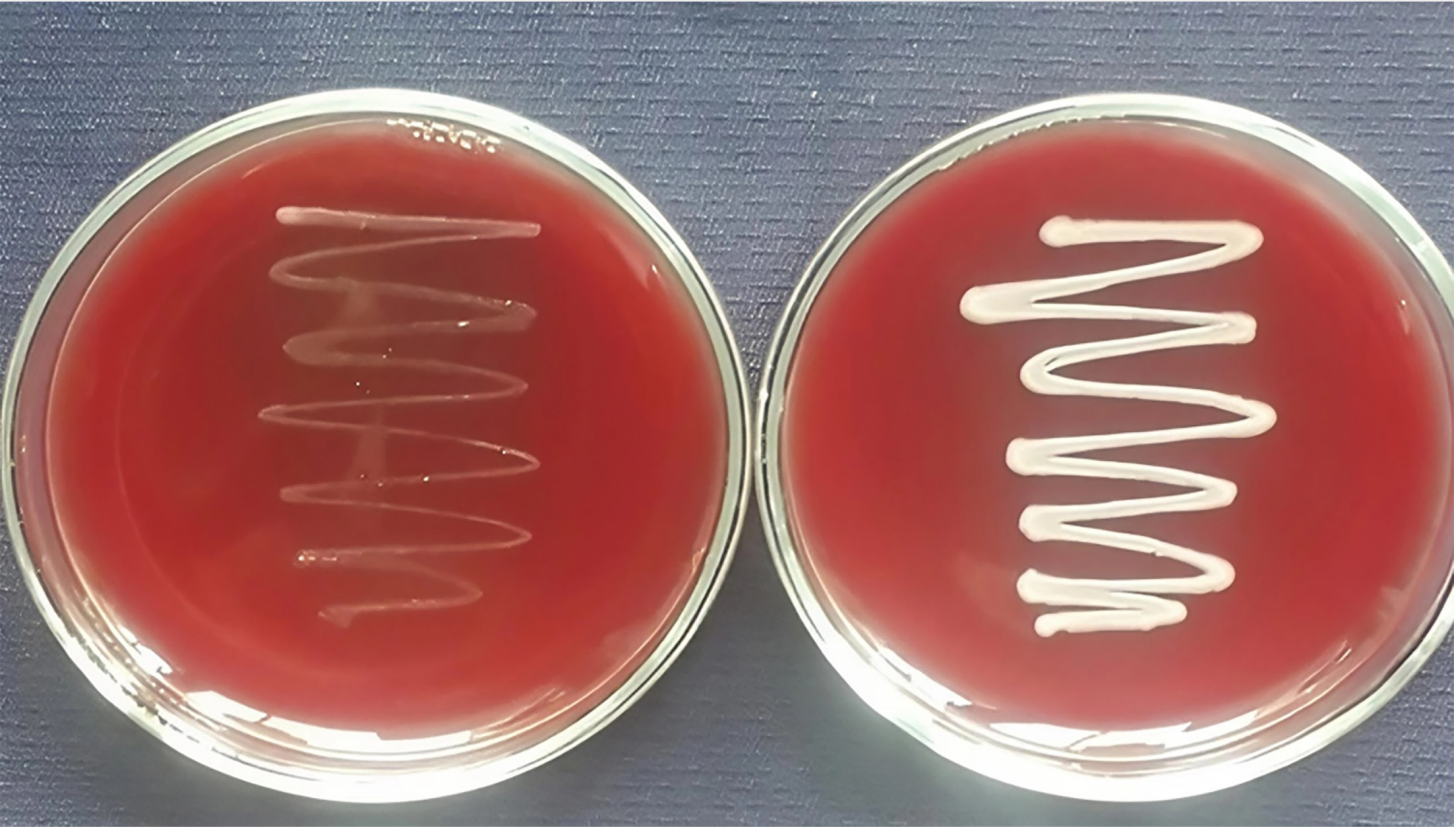
Bacterial growth observed on blood agar (37 °C for 24–48 h under aerobic conditions).

All 185 samples showed bacterial growth on appropriate culture media. Growth was observed on blood agar for a range of isolates (Fig. 1). All samples were also cultured on MacConkey agar, where Gram-negative bacteria were differentiated based on lactose fermentation characteristics, appearing as pink (lactose fermenters) or colourless colonies (non-fermenters).

### Modified string test results

**Fig. 2.**
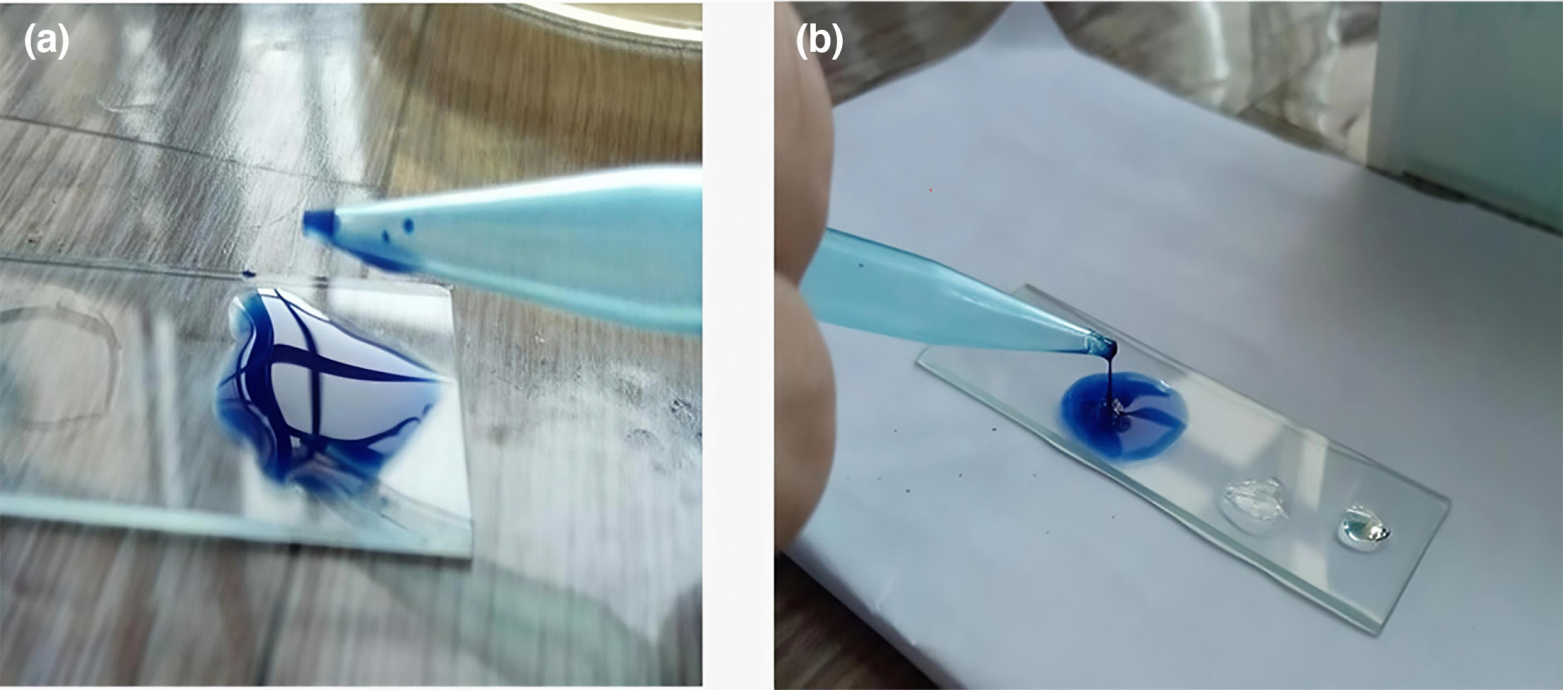
Modified KOH string test results. (**a**) Gram-positive isolate showing no string formation. (**b**) Gram-negative isolate showing visible string formation.

The modified KOH string test was performed on all isolates. All isolates classified as Gram-negative by Gram staining demonstrated visible string formation, whereas Gram-positive isolates showed no string formation. Representative results from environmental samples are shown in Figs2  3.

**Fig. 3.**
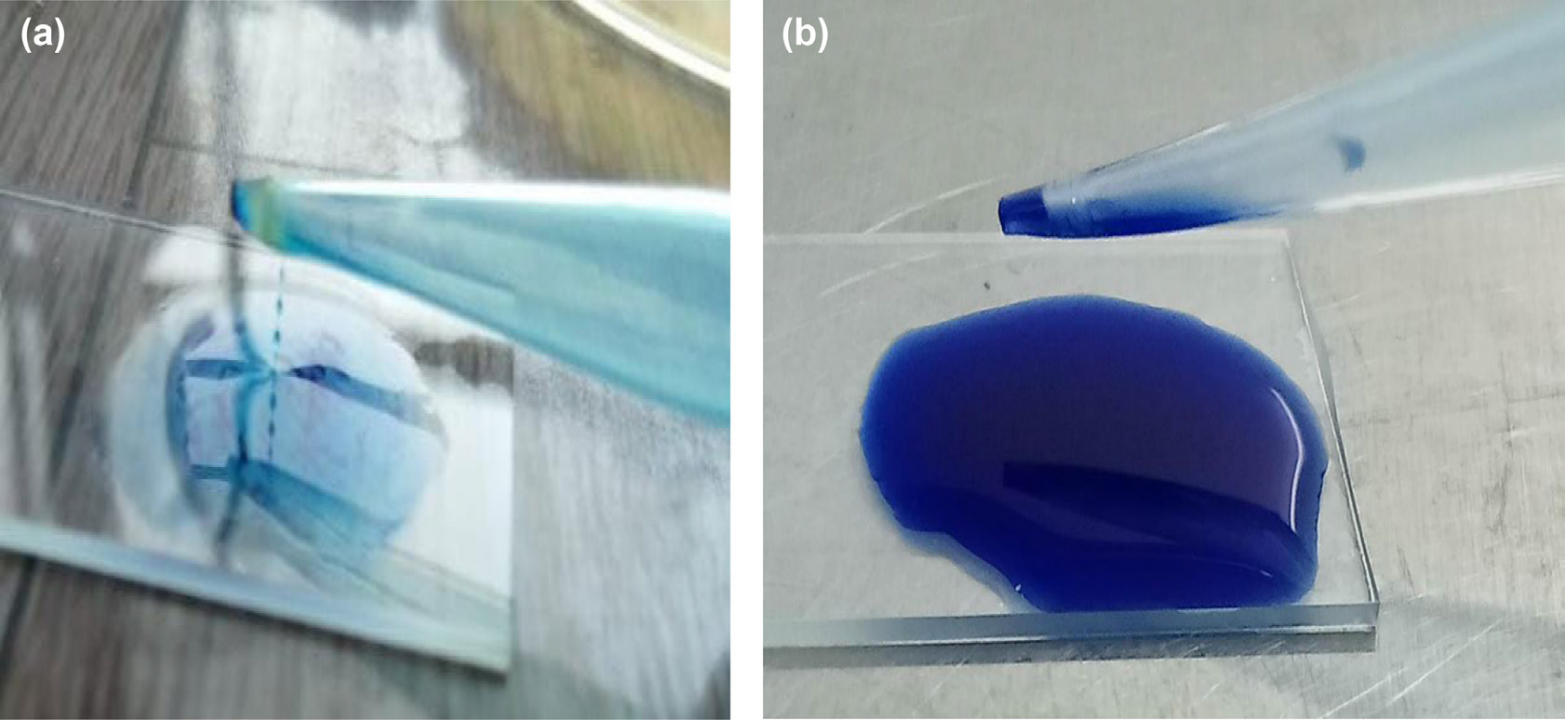
Modified KOH string test results for environmental samples. (**a**) Positive result showing visible string formation. (**b**) Negative result showing absence of string formation.

**Fig. 4.**
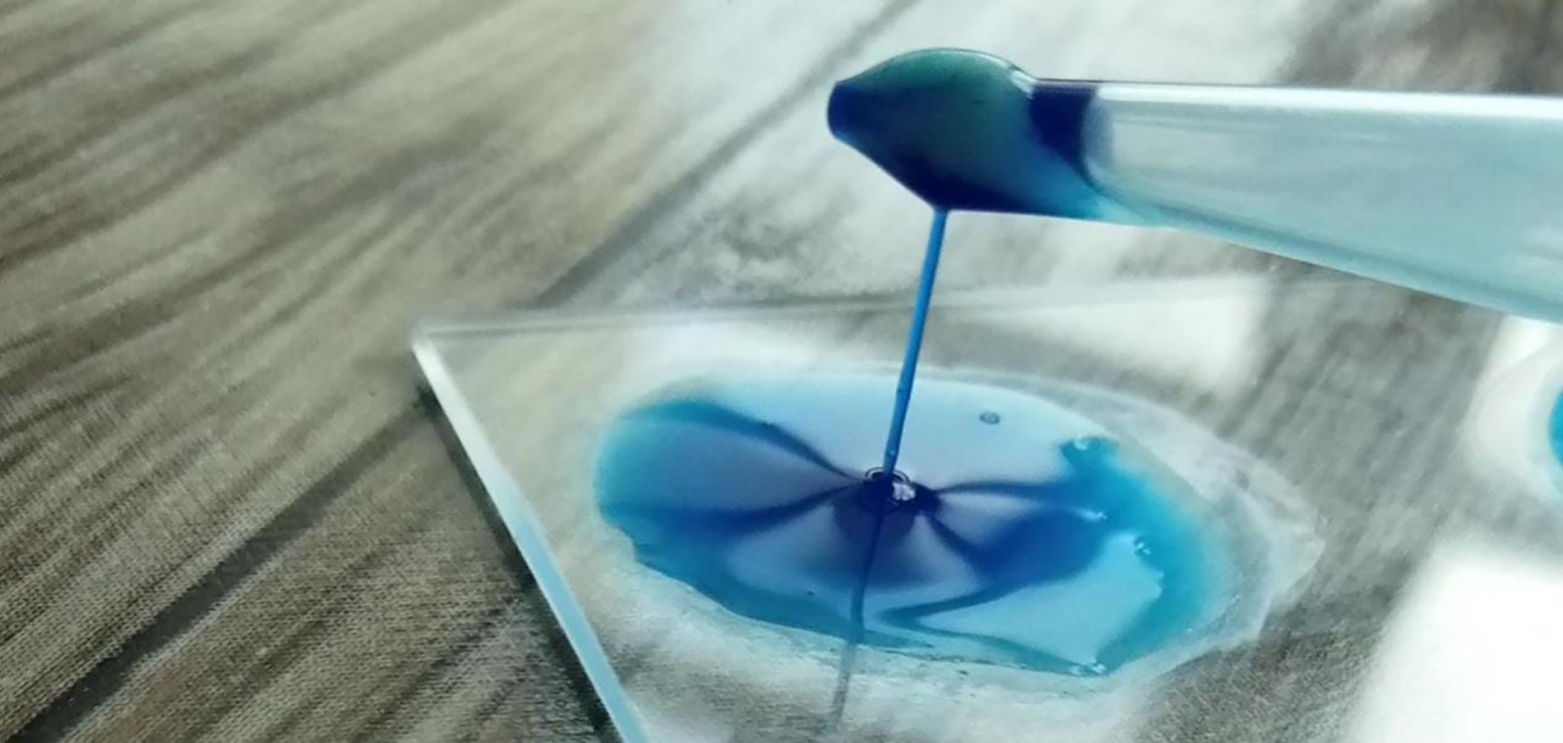
Visualization of string formation in clinical isolates following the addition of methylene blue.

The addition of methylene blue improved the visibility of filament formation, allowing clearer observation of string formation during the test. This effect was observed in clinical isolates, including *Klebsiella pneumoniae*, as shown in Fig. 4.

### Genotypic identification

A subset of isolates was subjected to 16S rRNA gene analysis following DNA extraction using a Cetyltrimethylammonium bromide based protocol). A total of ten isolates were randomly selected from environmental samples for 16S rRNA gene analysis to support taxonomic identification. The molecular analysis supported the taxonomic identification of the selected isolates.

## Discussion

The present study demonstrates that the incorporation of methylene blue into the KOH string test improves the visibility of filament formation, facilitating clearer interpretation of results. This observation is consistent with previous reports, highlighting the role of cationic dyes in binding to negatively charged DNA and enhancing visualization during bacterial lysis.

Our findings are in agreement with earlier studies, including Halebian *et al*. [14], which reported variability in the interpretation of the conventional KOH string test, particularly in cases with weak or ambiguous string formation. By improving visual contrast, the modified approach may help reduce ambiguity in test interpretation. In the present study, complete concordance was observed between the modified KOH test and Gram staining results within the tested sample set. In comparison to conventional methods, the modified KOH test offers a rapid and simple approach for preliminary differentiation of bacterial isolates. The procedure can be completed within a short time frame and requires minimal reagents and equipment. These characteristics make it particularly suitable for use in resource-limited settings or in situations where rapid screening is required. However, unlike Gram staining, the KOH test does not provide morphological information and is limited to differentiation based on cell wall properties. In resource-limited settings, where full Gram staining may not be feasible, Gram differentiation and morphological assessment may be performed as separate steps. The modified KOH string test can provide rapid Gram classification, while basic morphology may be assessed using simple microscopy with a single stain. This simplified workflow may reduce procedural complexity while retaining essential diagnostic information.

In addition to Gram staining, biochemical identification systems such as API are widely used for species-level identification. While these methods provide detailed metabolic profiling, they require longer incubation times and standardized laboratory conditions. In contrast, the modified KOH string test provides rapid preliminary differentiation within minutes, making it useful as an initial screening step rather than a replacement for biochemical identification. Additional dyes evaluated in this study (phenol red, safranin and iodine) showed limited enhancement in filament visibility (see Supplementary Material), supporting the selection of methylene blue as the most effective dye.

This study has several methodological limitations. The evaluation was not conducted under blinded conditions, and no standardized control strains were included, limiting the robustness of experimental validation. In addition, the performance of the modified KOH string test was not assessed for Gram-variable or atypical organisms, restricting the generalizability of the findings. Molecular confirmation using 16S rRNA gene analysis was performed on a limited subset of isolates and was not designed as a comprehensive validation framework. Furthermore, formal diagnostic performance metrics such as sensitivity and specificity were not calculated due to the absence of an independent gold standard and controlled study design.

Accordingly, the results should be interpreted as observed concordance within the tested dataset rather than validated diagnostic performance. The modified KOH string test should therefore be considered a complementary tool for rapid preliminary differentiation, rather than a replacement for established microbiological or molecular methods. Despite these limitations, the simplicity, rapid execution and minimal resource requirements of the method may offer practical advantages in resource-limited or high-throughput settings, where rapid initial classification is required.

## Conclusion

This study presents a modified KOH string test incorporating methylene blue to improve the visualization of filament formation during bacterial differentiation. The modified method showed complete concordance with Gram staining results within the tested sample set, with clear differentiation between Gram-negative and Gram-positive isolates. The incorporation of methylene blue enhances the visibility of the string reaction, facilitating easier interpretation without the need for specialized equipment. Due to its simplicity, low cost and rapid execution, the method may be particularly useful for preliminary bacterial differentiation in resource-limited or high-throughput settings. However, the modified KOH test does not replace conventional or molecular identification methods and should be considered as a complementary tool within a broader diagnostic workflow.

## Supplementary material

10.1099/acmi.0.000965.v3 Supplementary Material 1.

## Abbreviations

API: Analytical Profile Index
KOH: potassium hydroxide
