# Supplementary material for: Modified string test to improve and confirm by molecular characterization for bacterial identification
Source: Access Microbiol. 2026 Jun 25;8(6):000965.v3. doi: 10.1099/acmi.0.000965.v3 (PMC13293331; doi:10.1099/acmi.0.000965.v3)
Supplement: Supplementary Material 1. [file acmi-8-00965-s001.pdf]

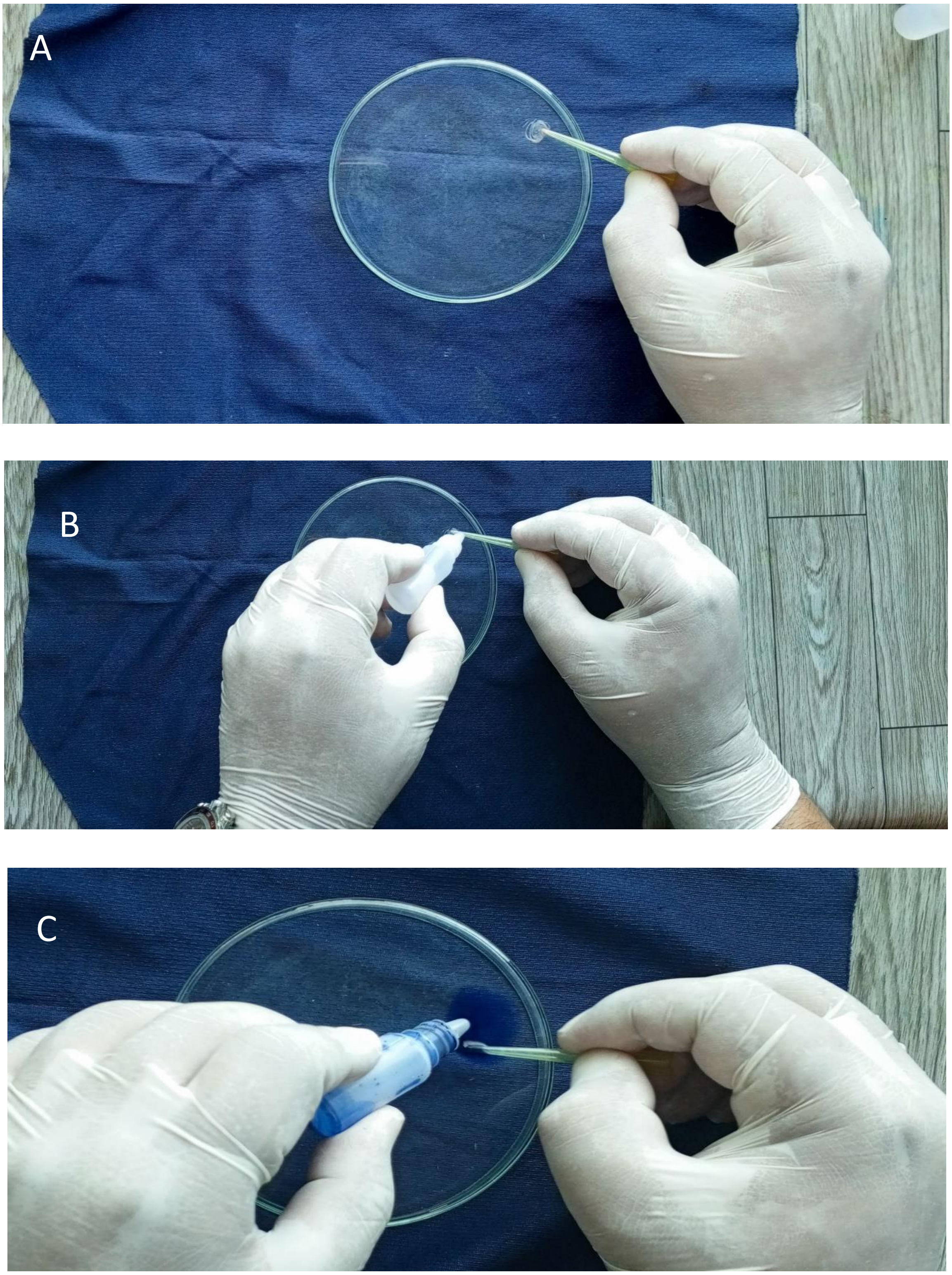

Supplementary Figure 1 This figure is showing the procedure of modified KOH string test, (a) emulsification of test organism, (b) addition of 3% KOH, (c) addition of 2% methylene blue.

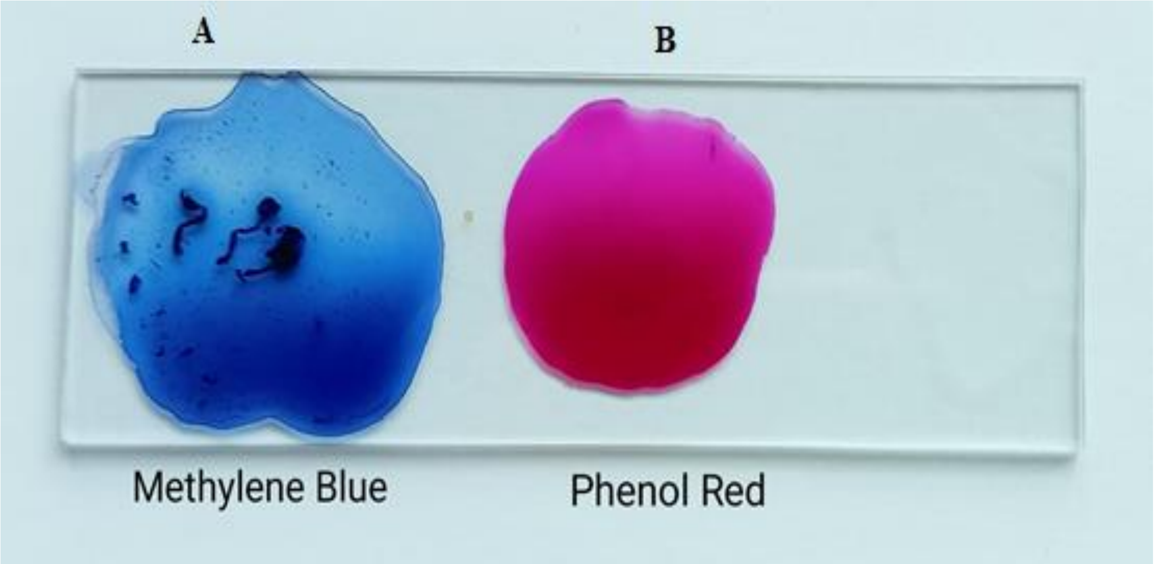

Supplementary Figure 2 Visualization of string produced during test using different dyes (A) Threads are visible on binding of methylene blue with DNA (B) No threads are not visible on binding of phenol red with DNA

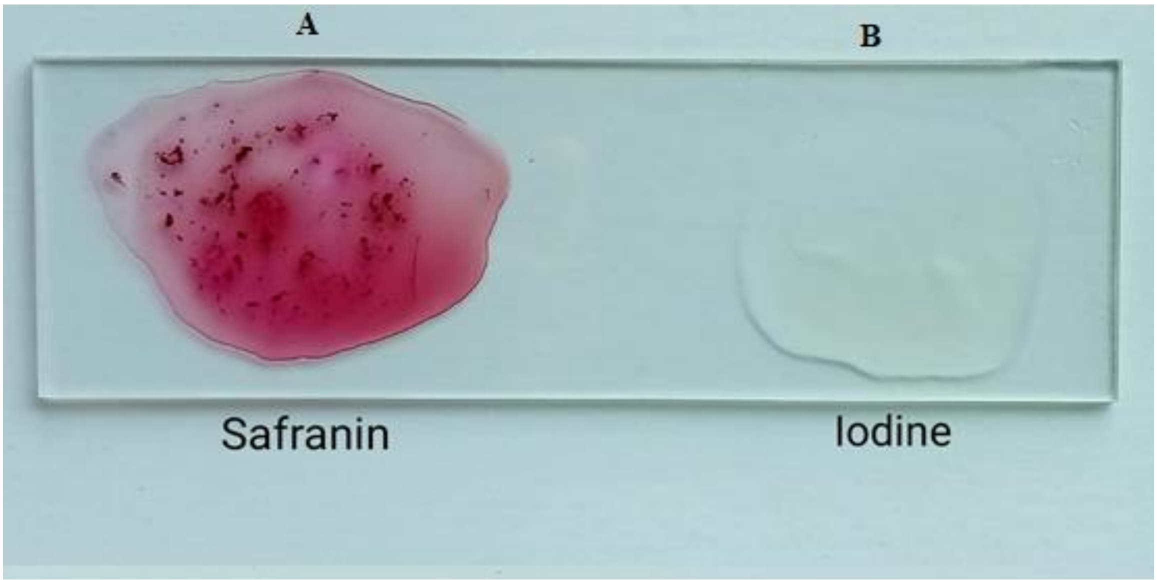

Supplementary Figure 3 (A) Safranin was used and similarly (B) Iodine was used in the experiment to check binding with DNA and improved visualization of string.

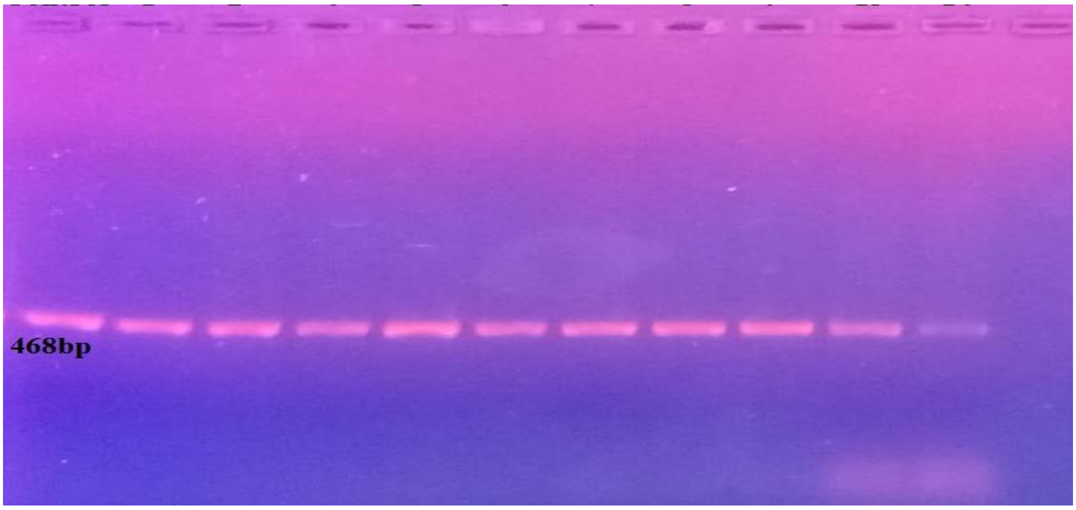

Supplementary Figure 4 PCR gel results showing DNA of 10 selected unknown bacteria with control of 468bp
